# Left Atrial Appendage Occlusion: Current Advances and Remaining Challenges

**DOI:** 10.1016/j.jacadv.2022.100136

**Published:** 2022-11-16

**Authors:** Mohamad Alkhouli, Christopher R. Ellis, Matthew Daniels, Megan Coylewright, Jens Erik Nielsen-Kudsk, David R. Holmes

**Affiliations:** a Department of Cardiology, Mayo Clinic School of Medicine, Rochester, Minnesota, USA; b Division of Cardiovascular Medicine, Department of Medicine, Vanderbilt University, Nashville, Tennessee, USA; c Institute of Cardiovascular Sciences, Core Technology Facility, University of Manchester, Manchester, United Kingdom; d Department of Cardiovascular Medicine, The Erlanger Heart and Lung Institute, Chattanooga, Tennessee, USA; e Department of Cardiology, Aarhus University Hospital, Aarhus N, Denmark

**Keywords:** anticoagulation, atrial fibrillation, left atrial appendage occlusion, stroke

## Abstract

The field of left atrial appendage occlusion is rapidly evolving. However, several issues remain including the limited randomized efficacy data, peri-device leak, device-related thrombus, and the ongoing refinement of procedural techniques. In this article, we provide a contemporary overview of left atrial appendage occlusion focusing on 4 key remaining challenges: efficacy data, peri-device leak, device-related thrombus, and procedural optimization.

## Highlights

• LAAO has emerged as a promising alternative to oral anticoagulation in selected patients with nonvalvular atrial fibrillation. Although remarkable progress in the field has been made, several questions remain open.
• This study summarized the remaining issues with LAAO including the need for more randomized data, device-related thrombus, peri-device leak, and procedural optimization.
• Ongoing randomized trials, newer devices, an improved planning software program, and procedural techniques will help address the remaining issues in the field.

Stroke prevention is a centerpiece in the management of atrial fibrillation (AF).^1^ Despite their efficacy in preventing ischemic strokes, anticoagulants are not utilized or not maintained in >50% of eligible patients due to bleeding risk, side effects, or noncompliance.^2^ Considering the growing size of the AF population and the substantial morbidity and mortality of AF-associated ischemic strokes, left atrial appendage occlusion (LAAO) has emanated as a feasible alternative to address these unmet needs.^3^ In the last decade, a wealth of data have emerged on the safety of LAAO accompanied with a rapidly growing adoption of the procedure in clinical practice.^4^ However, several issues remain including the limited randomized data demonstrating LAAO effectiveness and the concerns about device-related thrombus (DRT) and peri-device leak (PDL) and their management. In this article, we review the past and present of LAAO and provide a futuristic outlook of this rapidly evolving field focusing on the key remaining open questions (Central Illustration).

**Central Illustration:**
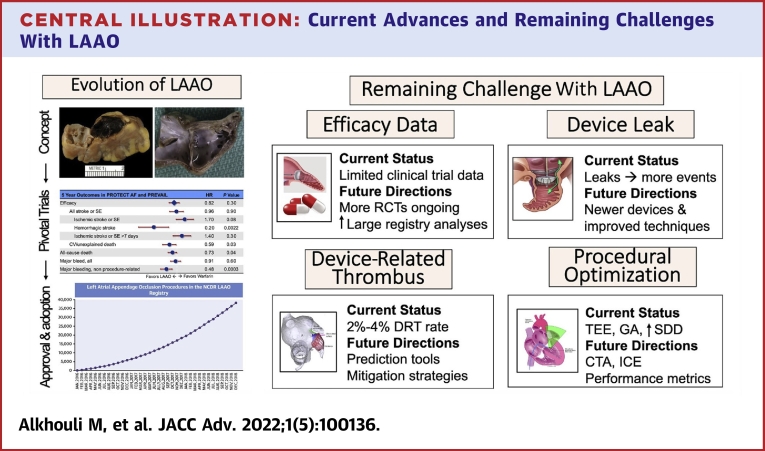
Current Advances and Remaining Challenges With LAAO CCT = cardiac computed tomography; DRT = device related thrombus; GA = general anesthesia; ICE = intracardiac echo; LAAO = left atrial appendage occlusion; RCT = randomized controlled trial; SDD = same day discharge; TEE = transesophageal echo.

## LAAO: past and present

**Figure 1:**
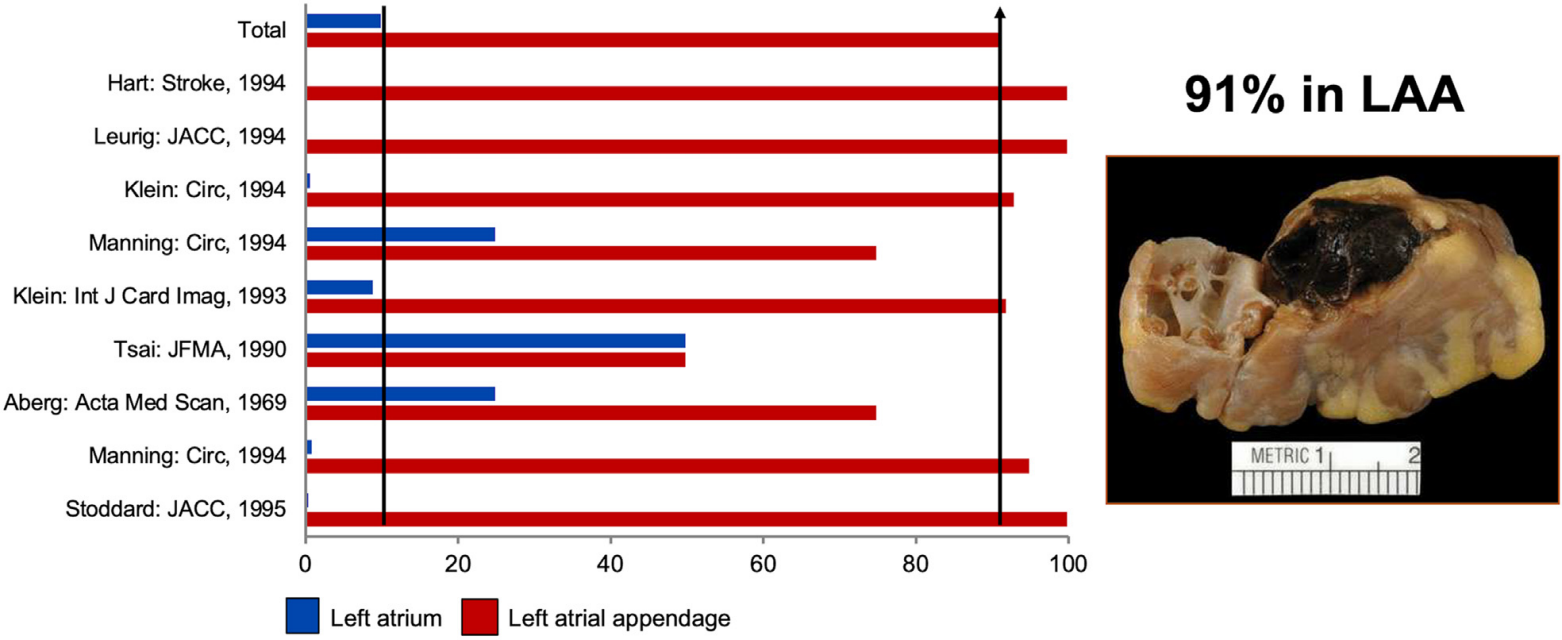
The Left Atrial Appendage as a Nidus for Thrombus Formation in Patients With Atrial Fibrillation LAA = left atrial appendage.

The concept of LAAO dates back to 1949 when John L. Madden reported the resection of the LAA in 2 patients for the “prophylaxis of recurrent thrombi.”^4^ Nonetheless, the interest in LAAO remained limited for decades until Blackshear and Odell published their seminal systematic review in 1996 that emphasized the potential role of the LAA as a nidus for thrombus in patients with nonvalvular AF (Figure 1).^5^ In the following years, surgical excision of the LAA at the time of concomitant cardiac surgeries became more popular albeit with wide variability in practice and virtually no supportive efficacy data.^6^ However, the emergence of the first transcatheter appendage occluder device in the early 2000 fueled major device innovation efforts and clinical investigations that subsequently led to the approval of percutaneous LAAO in the United States in 2015.

### Evidence supporting LAAO

Randomized data supporting the efficacy of LAAO have been limited. To date, only 3 randomized controlled trials (RCTs) comparing LAAO to anticoagulation have been published.^1^^,^^7^, ^8^, ^9^ A summary of the key findings of these studies is provided in Table 1. In the patient-level meta-analysis of PROTECT AF (WATCHMAN Left Atrial Appendage System for Embolic PROTECTion in Patients With Atrial Fibrillation) and PREVAIL (Evaluation of the WATCHMAN Left Atrial Appendage (LAA) Closure Device in Patients With Atrial Fibrillation Versus Long Term Warfarin Therapy) trials (1,114 patients) with a mean follow-up duration of 2.7 years, the primary efficacy endpoint (composite of stroke, systemic embolism, or cardiovascular or unexplained death) occurred with a similar frequency in both the device and control arms (HR: 0.82; 95% CI: 0.58-1.17; *P* = 0.27). The rate of ischemic stroke was higher in the device arm (1.6 per 100 patient-years vs 0.95 per 100 patient-years; HR: 1.71; 95% CI: 0.94-3.1; *P* = 0.08), counterbalanced by a lower rate of hemorrhagic stroke (HR: 0.2; 95% CI: 0.07-0.56; *P* = 0.002).^7^ These results remained largely similar in a subsequent patient-level meta-analysis with 5-year follow-up.^1^ PRAGUE (Left Atrial Appendage Closure vs. Novel Anticoagulation Agents in Atrial Fibrillation)-17 remains the only RCT to date that compared LAAO with direct oral anticoagulant (DOAC). The noninferiority of LAAO compared with DOAC documented in PRAGUE (Left Atrial Appendage Closure vs. Novel Anticoagulation Agents in Atrial Fibrillation)-17 was maintained in a subsequent analysis with 4 years of follow-up.^8^^,^^9^ The main limitation of the trial is that the noninferiority of LAAO was only powered for a composite endpoint that combined ischemic and bleedings events as well as procedural complications. The study was, however, underpowered to assess the impact of LAAO on lowering ischemic events, which is the presumed mechanism of action of the LAAO procedure.

**Table 1:** Key Findings of the LAAO Randomized Clinical Trials

Trial	Design	Patients	Key Findings
PROTECT AF (19683639)	Noninferiority Watchman vs warfarin	Device (n = 463) Control (n = 244)	• The primary efficacy endpoint (stroke, SE, and CV/unexplained death) event rate was 3.0 per 100 patient-years (95% CrI: 1.9-4.5) in the device group and 4.9 per 100 patient-years (2.8-7.1) in the control group (RR: 0.62, 95% CrI: 0.35-1.25) • Primary safety events were more frequent in the intervention group (7.4 per 100 patient-years, 95% CrI: 5.5-9.7 vs 4.4 per 100 patient-years, 95% CrI: 2.5-6.7; RR: 1.69, 1.01-3.19)
PREVAIL (24998121)	Noninferiority Watchman vs warfarin	Device (n = 269) Control (n = 138)	• First coprimary efficacy endpoint (stroke, SE, and CV/unexplained death) event rate at 18 mo was 0.064 in the device group vs 0.063 in the control group (RR 1.07 [95% CrI: 0.57-1.89])^a^ • Second coprimary efficacy endpoint (stroke or SE >7 d after randomization) was 0.025 vs 0.020 (risk difference 0.005 [95% CrI: −0.019 to 0.027]) • Adverse events lower than PROTECT AF (4.2% vs 8.7%; *P* = 0.004)
PRAGUE-17 (32586585)	Noninferiority LAAO device vs DOAC	Device (n = 201) Control (n = 201)	• The annualized rate of the primary composite outcome (stroke, TIA, SE, CV death, major or nonmajor clinically relevant bleeding, or procedure-/device-related complications) was 10.99% with LAAO and 13.42% with DOAC (sHR: 0.84; 95% CI: 0.53-1.31; *P* = 0.44; *P* = 0.004 for noninferiority) • Major LAAO-related complications occurred in 9 (4.5%) patients

CrI = credible interval; CV = cardiovascular; DOAC = direct oral anticoagulant; LAAO = left atrial appendage occlusion; PREVAIL = Evaluation of the WATCHMAN Left Atrial Appendage (LAA) Closure Device in Patients With Atrial Fibrillation Versus Long Term Warfarin Therapy); PRAGUE = Left Atrial Appendage Closure vs. Novel Anticoagulation Agents in Atrial Fibrillation; PROTECT AF = WATCHMAN Left Atrial Appendage System for Embolic PROTECTion in Patients With Atrial Fibrillation; RR = rate ratio; SE = systemic embolization; sHR = subdistribution hazard ratio; TIA = transient ischemic attack.

a Did not achieve the prespecified criteria noninferiority (upper boundary of 95% CrI ≥1.75).

**Figure 2:**
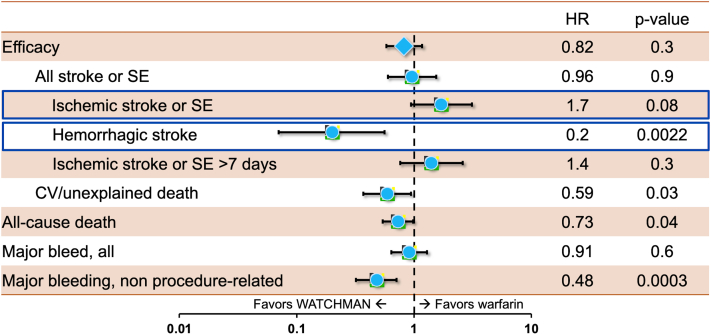
Patient-Level Meta-analysis Illustrating 5-Year Pooled Outcomes of PROTECT AF and PREVAIL Trials CV = cardiovascular; HR = hazard ratio; PREVAIL = Evaluation of the WATCHMAN Left Atrial Appendage (LAA) Closure Device in Patients With Atrial Fibrillation Versus Long Term Warfarin Therapy); PROTECT AF = WATCHMAN Left Atrial Appendage System for Embolic PROTECTion in Patients With Atrial Fibrillation; SE = systemic embolization.

The totality of the data suggests that LAAO is not inferior to anticoagulation in carefully selected patients with nonvalvular AF. However, several concerns remain. 1) A key issue with these data is the lack of convincing evidence that supports the mechanism of action of LAAO; reducing cardiac thromboembolism due to exclusion of the LAA cavity from systemic circulation. Indeed, ischemic events were higher in the device arm in PROTECT AF and PREVAIL (Figure 2), raising the question of whether LAAO is effective in eliminating the embolic source or whether its efficacy is merely driven by the mitigation of the bleeding risks associated with long-term anticoagulation. 2) The infrequency of ischemic events in the trial raises some concerns about the fragility of the conclusions. It is likely that the power calculations performed during trial design utilized expected ischemic rates based on historical risk-prediction schemes (ie, CHA2DS2-VASc) that are shown to overestimate the risk of ischemic stroke in contemporary practice.^10^ Hence, the potential need for larger trials to further confirm the role of LAAO for stroke prevention has been raised.^11^ 3) These trials only enrolled patients who are deemed candidate for a short-term course of anticoagulation after the procedure. No randomized data are yet available to support LAAO in patients with absolute contraindication to anticoagulation. The only RCT that was designed to assess this population (ASAP-TOO [Assessment of the WATCHMAN™ Device in Patients Unsuitable for Oral Anticoagulation] trial; NCT02928497) was terminated due to enrollment difficulties although follow-up for the enrolled patients will continue through 5 years.

Numerous nonrandomized studies have documented the efficacy of LAAO in reducing ischemic stroke and major bleeding.^12^ However, these studies were observational, used heterogenous endpoints, lacked a control arm, and indirectly derived efficacy conclusions by comparing the ischemic and bleeding event rate with what is predicted by the CHA2DS2-VASc and HASBLED scores, respectively. Therefore, although these data provided reassurance, it did not generate the level of evidence needed to widely accept LAAO as a mainstream stroke-prevention modality, and the need for further randomized data remain.

### FDA approval and guidelines

The congregate data from PROTECT AF, PREVAIL, and their respective registries led to the approval of LAAO with the Watchman 2.5 (Boston Scientific) device by the Food and Drug Administration (FDA) in 2015. In 2021, a second LAAO device (Amulet, Abbott) was approved by the FDA based on its noninferiority to the Watchman 2.5 device.^13^ The Amulet investigational device exemption trial, the largest published LAAO trial to date, randomized 1,878 patients to LAAO with the Watchman 2.5 device or the Amulet occluder. The Amulet occluder was noninferior to the Watchman device for the primary effectiveness endpoint (composite of ischemic stroke or systemic embolism at 18 months, 2.8% vs 2.8%; *P* < 0.001 for noninferiority) and for the composite of stroke, systemic embolism, or cardiovascular/unexplained death (5.6% vs 7.7%; *P* < 0.001 for noninferiority). Although professional societies eventually incorporated LAAO in their guidelines, their recommendations for LAAO are weak (Class IIb, Level of Evidence: C) and critical of the lack of robust evidence supporting LAAO^14^^,^^15^ (Table 2). In addition, to ensure the rational dispersion and continuous safety of the procedure, the FDA required LAAO programs to adopt a shared decision-making process involving nonimplanting physicians and decision-aid tools and to participate in a national registry for ongoing surveillance of clinical outcomes.

**Table 2:** Current U.S. and European Guidelines on the Use of Percutaneous LAAO Devices

Society, Year	COR	LOE	Recommendation
AHA/ACC/HRS, 2019	IIb	B-NR	Percutaneous LAA occlusion may be considered in patients with AF at increased risk of stroke who have contraindications to long-term anticoagulation (Clinical trial data and FDA approval of the Watchman device necessitated this recommendation.)
ESC/EACTS, 2020	IIb	B	LAA occlusion may be considered for stroke prevention in patients with AF and contraindications for long-term anticoagulant treatment (eg, intracranial bleeding without a reversible cause)

ACC = American college of cardiology; AF = atrial fibrillation; AHA = American heart association; COR = class of recommendation; EACTS = European Association for Cardio-Thoracic Surgery; ESC = European Society of Cardiology; FDA = Food and Drug Administration; HRS = Heart Rhythm Society; LAA = left atrial appendage; LOE = level of evidence.

### Utilization rates and safety data

**Figure 3:**
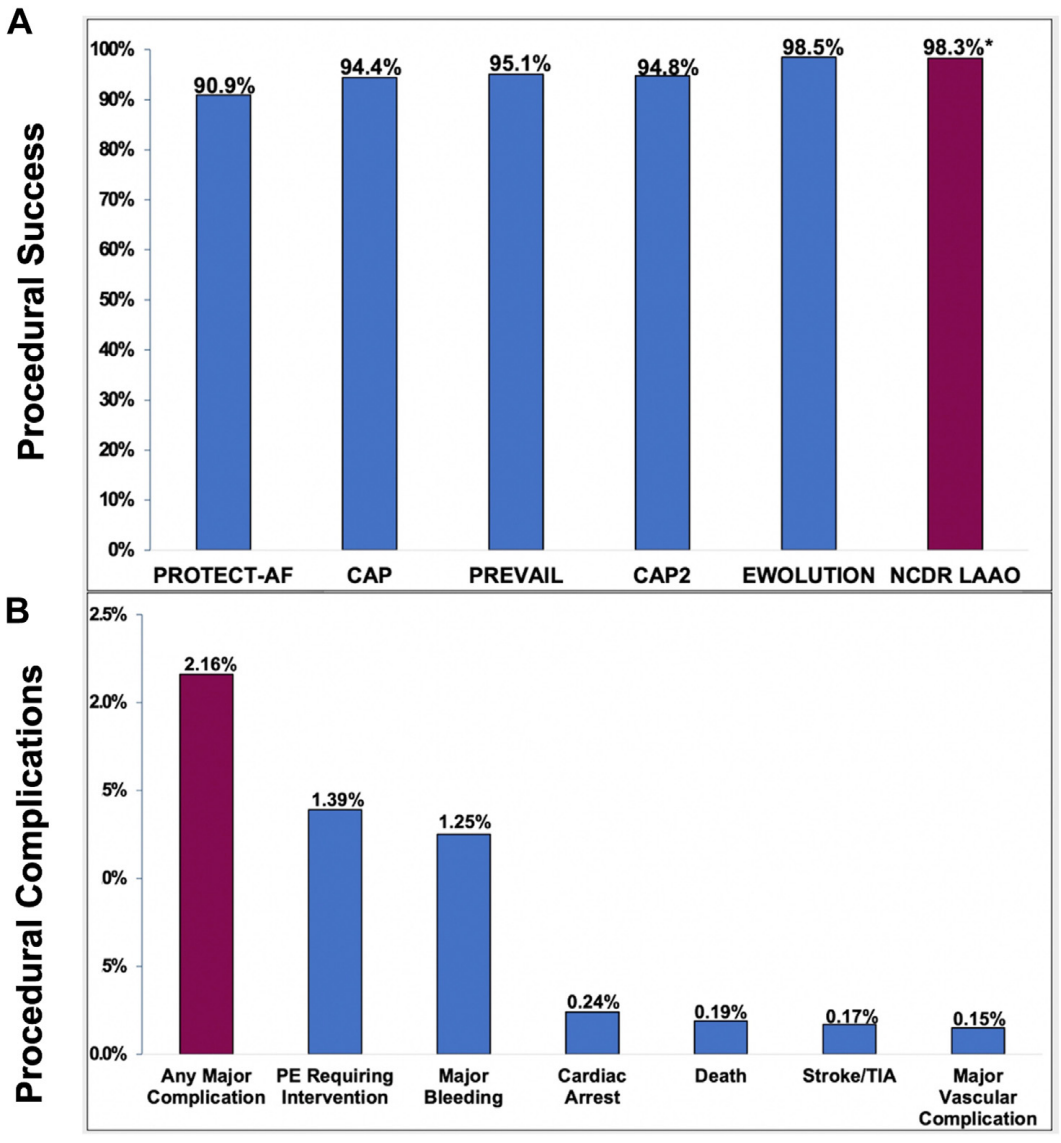
Procedural Outcomes in the NCDR LAAO Registry **(A)** Procedural success in LAAO Registry compared with the early LAAO trials and their nested registries. **(B)** Procedural complications in the LAAO Registry. CAP = continuous access to PROTECT AF registry; EWOLUTION = Registry on WATCHMAN Outcomes in Real-Life Utilization); PE = pericardial effusion; TIA = transient ischemic attack.

Following FDA approval, the utilization rates of LAAO in the U.S. grew substantially, and the safety profile of the procedure remained excellent. Indeed, the success rates recorded in national registries were higher, and the complication rates were lower than those reported in the pivotal trials and their nested registries (Figure 3).^16^, ^17^, ^18^, ^19^, ^20^, ^21^ In addition to providing reassurance regarding the safety of LAAO in commercial settings, a survey of the initial experience with LAAO in the US reveals other important observations:

1. Off-label practices: LAAO for an off-label indication was not uncommon. For example, 14% of patients receiving LAAO in the US had atrial flutter and not AF, a population that was not studied in the RCT.^17^ In addition, operators frequently used an off-label post-thrombotic regimen. For example, the instructions for use of the Watchman 2.5 device require patients to remain on warfarin for 45 days after the procedure. However, only 51% of patients who received the device in the US between 2016 and 2018 were discharged on warfarin.^22^
2. Disparities in LAAO utilization: Most patients who undergo LAAO in the U.S. were of White race. In the LAAO registry, Black and Hispanic patients represented only 4.6% and 0.6% of patients, respectively.^17^^,^^23^
3. Disparities in LAAO outcomes: Although women have been shown to be at higher risk of major complications after various cardiovascular interventions, the magnitude of difference in outcomes between men and women after LAAO is considerably higher. Data from the LAAO registry and from the national readmission database showed a 2-fold increase in major adverse events with LAAO in women compared with men.^24^^,^^25^ Similarly, Black and Hispanic patients experienced 30% and 90% higher rates of in-hospital complications, respectively, after LAAO than White patients.^26^
4. Differential impact of device type on safety outcomes: The rate of in-hospital pericardial effusion with the Watchman FLX device was 2.37%, of which ∼50% were treated with percutaneous drainage, and 11.4% required a cardiac surgery.^21^ This was substantially reduced with the second-generation Watchman FLX device with which the rate of pericardial effusion requiring intervention was only 0.42%.^27^
5. Current data in the U.S. pertain only to the Watchman FLX device and its predecessor; Watchman 2.5. Postmarket outcome data with the recently approved Amulet device in the U.S. are not yet available.

## Efficacy data for LAAO

PROTECT AF and PREVAIL paved the way for regulatory approval of LAAO in the U.S. in 2015. However, societal guidelines on AF management highlight the need for more randomized data supporting the efficacy of LAAO.^14^^,^^15^ Several prospective trials have been commenced to address this need. A summary of the trials, their objective, and their characteristics is shown in Tabel 3. The results of these trials will be essential to further validate the efficacy of the LAAO concept overall and to assess its role in low-risk patients as well as in special population (eg, patients with contraindication to anticoagulation, patients with aortic stenosis undergoing transcatheter aortic valve replacement, and patients undergoing catheter ablation for AF). Other prospective trials that are being currently considered pertain to the emerging concept of combining LAAO with anticoagulation to achieve optimal stroke prevention. This concept stems from recent randomized data (LAAOS III trial) that demonstrated the superiority of surgical LAA closure along with anticoagulation to anticoagulation alone in AF patients undergoing a cardiac surgery.^28^ In this trial, patients who underwent LAA closure had a 33% relative risk reduction of ischemic stroke or systemic embolization compared with those treated with anticoagulation alone (HR: 0.67; 95% CI: 0.53-0.85; *P* = 0.001).^28^ Although this approach would be only limited to patients eligible for long-term anticoagulation, a proof-of-concept observational study recently showed that results after adding a reduced dose of oral anticoagulant to LAAO are superior to those with LAAO alone suggesting that a tailored combination therapy might be feasible even in high-bleeding-risk patients.^29^

**Table 3:** Overview of Current and Planned Randomized Trials on Percutaneous LAAO

Study Name/Sponsor	Trial Size	Trial Objective	Intervention	Control	Primary Outcome Measures	Follow-Up
CHAMPION-AF (NCT04394546) Boston Scientific	3,000	Assess the role of LAAO in NVAF patients who are eligible for long-term DOAC	LAAO with Watchman/FLX	DOAC	Composite of ischemic stroke or SE; Composite of ischemic stroke, SE, or CV death (NI); nonprocedural major bleeding (S)	3 y
CATALYST (NCT04226547) Abbott	2,650	Assess the role of LAAO in NVAF patients who are eligible for long-term DOAC	LAAO with Amulet	DOAC	Composite of ischemic stroke or SE; Composite of ischemic stroke, SE, or CV death (NI); nonprocedural major bleeding (S)	3 y
OCCLUSION-AF (NCT03642509) Aarhus University	750	Assess the role of LAAO in NVAF patients who are eligible for long-term DOAC	LAAO with Amulet or Watchman	DOAC	Composite of stroke, SE, major bleeding, and all-cause mortality	5 y
CLOSURE-AF (NCT03463317) Charite University	1,512	Assess the role of LAAO in NVAF patients with high bleeding risk or contraindication to OAC	CE-mark/approved LAAO device	DOAC or VKA	Composite of stroke, SE, major bleeding (BARC type 3-5), CV, or unexplained death	2 y
STROKECLOSE (NCT02830152) Nordic Universities	750	Assess the role of LAAO in NVAF patients with an ICH within 12 mo	LAAO with Amulet	Medical therapy	Composite of stroke, SE, major bleeding, and all-cause mortality	5 y
CLEARANCE (NCT04298723) Jena University	550	Assess the role of LAAO in NVAF patients with a history of ICH	LAAO with Watchman FLX	Medical therapy	Composite of stroke, SE, BARC type 2-5 bleeding, and CV or unexplained death	2 y
COMPARE-LAAO (NCT04676880) R&D Cardiologie	609	Assess the role of LAAO in NVAF patients with contraindication for OAC	LAAO with Watchman FLX or Amulet	Antiplatelets or no therapy	Time to first occurrence of stroke; Time to first occurrence of the stroke, TIA, or SE; Procedural complications	5 y
OPTION (NCT03795298) Boston Scientific	1,600	Assess the role of LAAO in NVAF patients undergoing catheter ablation for AF	LAAO with Watchman/FLX	DOAC	Composite of stroke, death, or SE (NI); nonprocedural major bleeding (S)	3 y
WATCH-TAVR (NCT03173534) Boston Scientific	350	Assess the role of LAAO in NVAF patients undergoing TAVR	TAVR + LAAO with Watchman	TAVR + medical therapy	All-cause mortality, stroke, and bleeding	1 y
CONFORM^a^ (NCT05147792) Conformal Medical	1,400	Assess the performance of the CLAAS device (head-to-head device trial)	LAAO with CLASS device	LAAO with Watchman FLX or Amulet	Procedure-related complications, all-cause death, major bleeding (12 mo); ischemic stroke or SE (18 mo)	1.5 y
WAVECREST2^a^ (NCT03302494) Coherex Medical	1,550	Assess the performance of the WaveCrest device (head-to-head device trial)	LAAO with WaveCrest	LAAO with Watchman	Procedure-related complications (45 d), all-cause death; major bleeding; ischemic stroke or SE (24 mo)	2 y

BARC = Bleeding Academic Research Consortium; CE = Conformite Europeenne; CLAAS = Conformal; CV = cardiovascular; DOAC = direct oral anticoagulant; ICH = intracranial hemorrhage; LAAO = left atrial appendage occlusion; NI = noninferiority; NVAF = nonvalvular atrial fibrillation; OAC = oral anticoagulation; S = superiority; SE = systemic embolization; TAVR = transcatheter aortic valve replacement; TIA = transient ischemic attack; VKA = vitamin-K antagonist.

a Active, not yet recruiting.

## Device-related thrombus

Thrombus formation on LAAO devices has been a subject of major concern.^30^ Numerous studies have documented the incidence of DRT, its timing, and its association with adverse events. Fewer studies have investigated the predisposing factors to DRT and the effectiveness of its various management strategies.

### Frequency and timing of DRT

The incidence of DRT in PROTECT AF, PREVAIL, and their nested continuous access registries was 3.74%.^31^ Notably, one-third of DRT cases were detected at the time of unplanned transesophageal echocardiograms (TEEs). In a meta-analysis including >10,000 patients, the pooled incidence of DRT was 3.8%.^32^ In this meta-analysis, the diagnosis was made in <90, 90 to 365, and >365 days in 42%, 57%, and 1% of patients, respectively. In the Amulet IDE trial, the incidence of DRT at 18 months was 3.3% in the Amulet arm and 4.5% in the Watchman arm.^13^ In a prospective registry with the second-generation Watchman FLX devices, the DRT rate was 1.7% at 1 year with 3 of 7 cases detected beyond 300 days after the procedure.^33^

### Clinical significance of DRT

The association of DRT with thromboembolic events is well established. In the pivotal Watchman trials, 26.2% of patients with DRT experienced a stroke or systemic embolism event within 6 months of DRT detection.^31^ In a global dedicated DRT registry, DRT was associated with >3-fold increase in the risk of ischemic stroke (HR: 3.49; 95% CI: 1.35-9.00; *P* = 0.01).^34^ In the EURO-DRT (European-Canadian device related thrombus registry) registry, the incidence of stroke and death at 2 years among patients with DRT was 13.8% and 20%, respectively.^35^ In a meta-analysis of 66 studies, the incidence of ischemic stroke was 13.2% in patients with DRT vs 3.8% in patients without DRT (odds ratio: 5.27; 95% CI: 3.66-7.59; *P* < 0.001).^32^

### Risk factors for DRT

**Figure 4:**
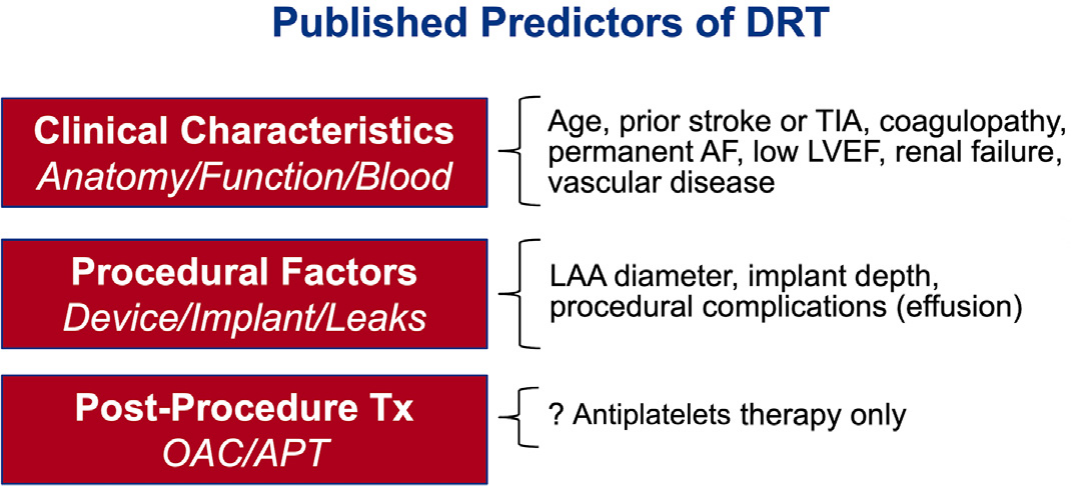
Predictors of DRT in the LAAO Literature AF = atrial fibrillation; APT = antiplatelet therapy; DRT = device-related thrombus; LAA = left atrial appendage; LVEF = left ventricular ejection fraction; OAC = oral anticoagulation; TIA = transient ischemic attack.

**Figure 5:**
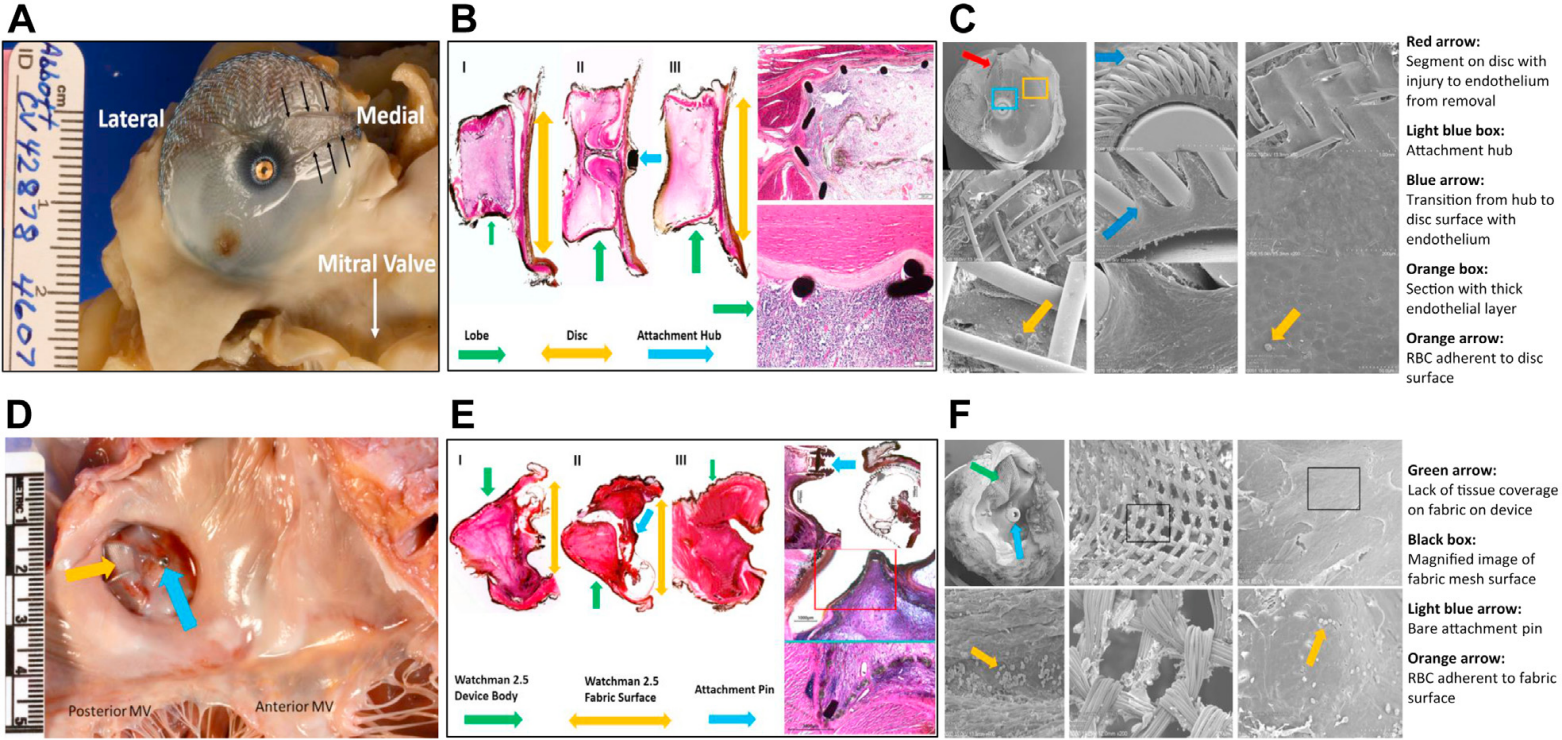
Endothelialization after LAAO With Different Occluders Shown are the Amulet Occluder **(A to C)** and Watchman 2.5 Device **(D to F)**. **(A and D)** Gross inspection showing both devices properly positioned. **(B and E)** Microscopic inspection using hematoxylin and eosin stain showing both devices with complete left atrial appendage cavity fibrosis and seal with no peridevice leak. **(C and F)** Scanning electron microscopy showing bare components of both devices with exposed fabric and/or exposed attachment hubs. Disruption of tissues covering the device during explant sloughed off neoendothelium on the Amulet Occluder, as the edge of the neoendothelium is not tapered and has sharp demarcations **(C**, **red arrow)**. Reprinted with permission from Ellis et al. *J Am Coll Cardiol EP*. 2022;8(6):828-829. https://doi.org/10.1016/j.jacep.2022.01.024. MV = mitral valve; RBC = red blood cells.

Identifying predisposing factors for DRT is crucial to optimize risk stratification and procedural outcomes. However, this task has been challenging due to the large number of potential risk factors and the low DRT event rate overall. Nonetheless, several predictors of DRT have been identified in the literature (Figure 4).^30^^,^^34^ One study attempted to model a risk-prediction scheme (the DRT score) to provide a practical aid for clinicians when considering patients for LAAO. The DRT score was derived from an international registry of 711 patients (237 with and 474 without DRT).^34^ In this registry, among >40 candidate risk factors considered in the logistic regression model, 5 were independently predictive of DRT (hypercoagulopathy, renal insufficiency, permanent AF, deep device implantation, and pericardial effusion). Although the type of post-LAAO antithrombotic therapy in this global registry did not impact the risk of DRT, other studies yielded opposite conclusions. In the Watchman trials and nested registries, the incidence of DRT was higher when the post-LAAO regimen included antiplatelets therapy alone vs anticoagulation (3.1% vs 1.4%, *P* = 0.018).^36^ In the NCDR LAAO (National Cardiovascular Data Registry Left Atrial Appendage Occlusion) registry, a short course of anticoagulation with warfarin or a DOAC after the procedure was associated with a lower incidence of major adverse events through 6 months of follow-up.^22^ Finally, whether the risk of DRT is device-specific remains uncertain. The Amulet device had a slightly lower DRT rate than the Watchman 2.5 device in the Amulet IDE trial, and this was hypothesized to be related to the larger neo-LAA that remains with plug-based vs disc-lobe device and to the differential impact on device design on healing and endothelialization (Figure 5).^13^^,^^37^^,^^38^ This concept remains to be corroborated in further studies.

**Figure 6:**
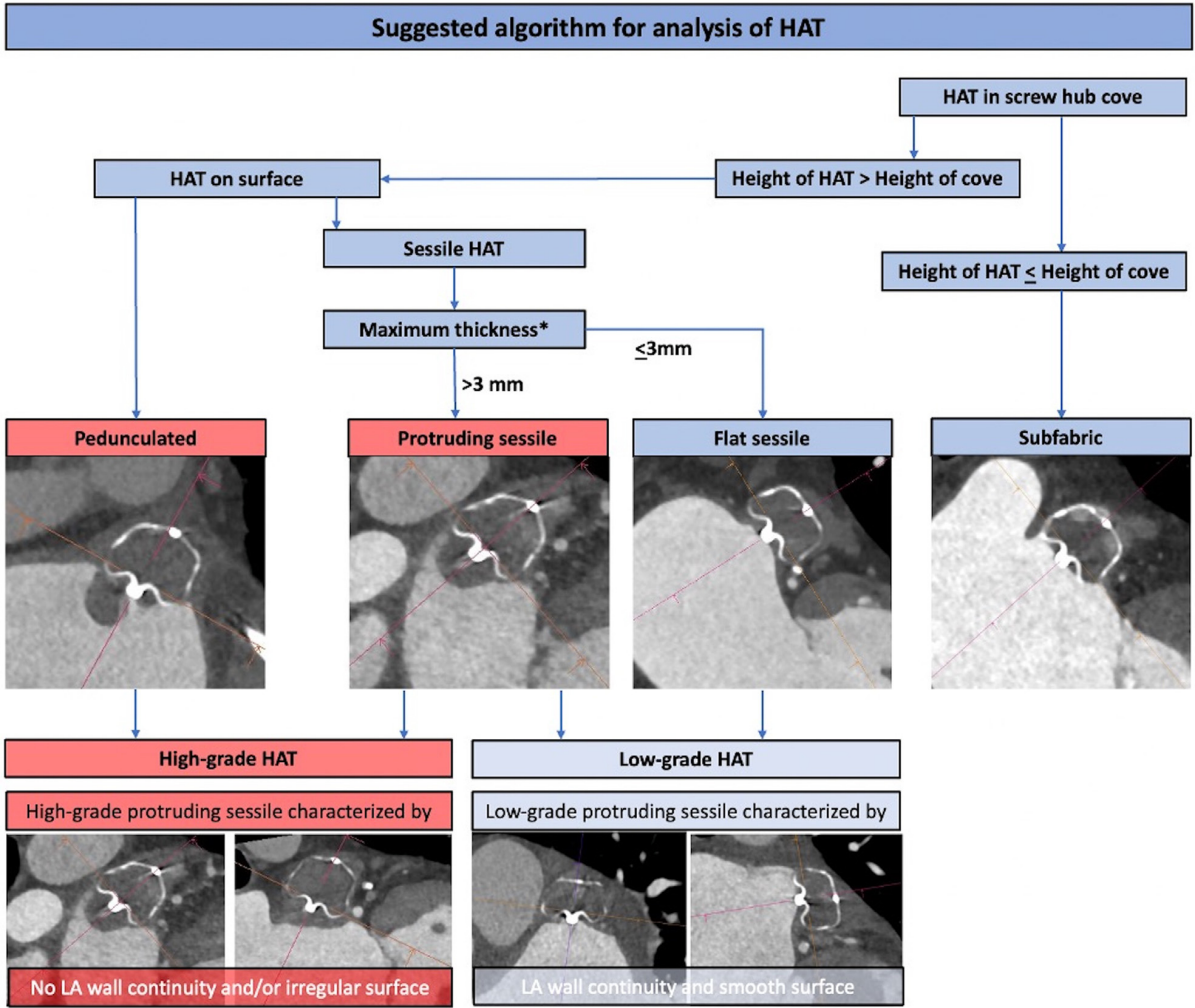
Suggested Algorithm for Assessment of Device Thrombus and Hypoattenuating Thickening After LAAO With the Watchman FLX Device HAT = hypoattenuating thickening.

Emerging concepts in DRT prediction include the potential role of flow dynamics on thrombus formation and the early detection of DRT precursors using cardiac computed tomography (CCT). Mill et al^39^ reported a proof-of-concept use of computational modeling to potentially predict DRT based on flow dynamic patterns. Using a web-based interactive virtual implantation platform, Aguado et al^40^ showed that computational flow dynamic simulations may be able to predict the most appropriate LAAO configurations (type of device, size, landing zone) for a given patient-specific LAA morphology to reduce the risk of DRT. A multicenter collaborative study is currently underway to further explore this concept with preliminary data showing promising results. The growing use of CCT in post-LAAO surveillance also afforded a unique opportunity to further understand the patterns of DRT on contemporary LAAO devices. In a recent study by Kramer et al,^41^ the authors assessed the frequency and phenotypes of hypoattenuated thickening (HAT) observed on CCT after LAAO with the Watchman FLX device. Although the study was not powered for correlation of different HAT patterns with clinical events, it was the first study to propose a framework for assessing normal device healing vs the various patterns of HAT/DRT after LAAO^41^ (Figure 6).

### Treatment of DRT

The management of DRT continues to represent a clinical conundrum. Although some studies suggested that oral or parenteral anticoagulants are effective in resolving DRT in majority of patients, several issues remain. First, most patients referred for LAAO are not suitable candidates for intensified or prolonged anticoagulation regimens and may therefore be left with 2 opposing high-risk scenarios (risk of embolic events with DRT vs risk of major bleeding with resumption or initiation of anticoagulation). Second, even among patients treated with anticoagulation, DRT persists in 20% to 25% of them, and they experience substantially higher morbidity and mortality.^34^^,^^35^ Third, even when DRT is resolved with anticoagulation, recurrence rates are high (35% while still on anticoagulation, and 50% when anticoagulation is stopped).^42^ Finally, not all DRTs are the same, and the management of large and/or highly mobile thrombi remains uncertain. The feasibility of transcatheter aspiration of DRT has been reported, but the safety and efficacy of this approach for the routine management of high-risk DRTs has not been established.^43^ Iterative LAAO device designing also considered the risk of DRT. For example, the Watchman FLX device has significantly less exposed metal screw on the surface of the device to reduce the risk of DRT. Device manufactures are also exploring novel preventative methods of DRT such as the addition of antithrombotic device coating to minimize the risk of thrombus formation on the device akin to what has been used with drug-coated stents.

## Peri-device leak

The proposed mechanism of action of LAAO is that the exclusion of the trabeculated LAA tissue from the systemic circulation will lead to a lower risk of thromboembolic events as the LAA is the source of thrombi in most patients with nonvalvular AF. However, there is ample evidence now that percutaneous LAAO devices frequently do not achieve “complete occlusion” of the appendage, raising the question of whether the term “occlusion” is indeed a misnomer.

### Frequency of PDL

The incidence of PDL varies considerably due to the lack of consensus on leak detection and classification methodology. Furthermore, the cutoff for what is considered a potentially significant leak differs across studies. Nonetheless, the literature suggests a high incidence of PDL after LAAO, with higher rates reported in RCTs with core lab adjudication than in observational registries.^44^ In PROTECT AF, any PDL was present in 40.9% of patients at 45 days, which decreased to 32.1% at 1 year.^45^ Leaks >3 mm in diameter were present in 13.3% at 45 days and in 11.8% at 1 year. In the Amulet IDE trial, any PDL at 45 days was present in 37% and 54% of patients randomized to the Amulet vs Watchman device, respectively.^13^ In addition, leaks >3 mm in diameter were detected in 10% and 25% of patients in the Amulet vs Watchman arms, respectively. In a large real-world study including 51,333 patients enrolled in the NCDR LAAO registry, any PDL was documented in 26.6% of patients at 45 days.^16^ All the abovementioned studies included patients treated with the first-generation Watchman 2.5 device. The newer Watchman FLX device has not been assessed in a randomized trial. However, data from the prospective PINNACLE (Protection Against Embolism for Nonvalvular AF Patients: Investigational Device Evaluation of the Watchman FLX LAA Closure Technology) registry (n = 400) adjudicated by an echocardiography core lab suggested a lower incidence of PDL with the FLX device (any PDL 17.4% at 45 days and 10.5% at 1 year).^33^ A large portfolio of LAAO devices are being currently evaluated in preclinical and early feasibility studies. Whether these devices will offer an incremental advantage over the Amulet and Watchman FLX devices with regards to attaining a complete seal of the LAA remains to be seen. Preliminary data with a novel foam-based conformable device (Conformal, Conformal Medical Inc) suggest that the device is effective in achieving a complete seal of the LAA in ∼94% of patients.^46^

### Clinical impact of PDL

**Figure 7:**
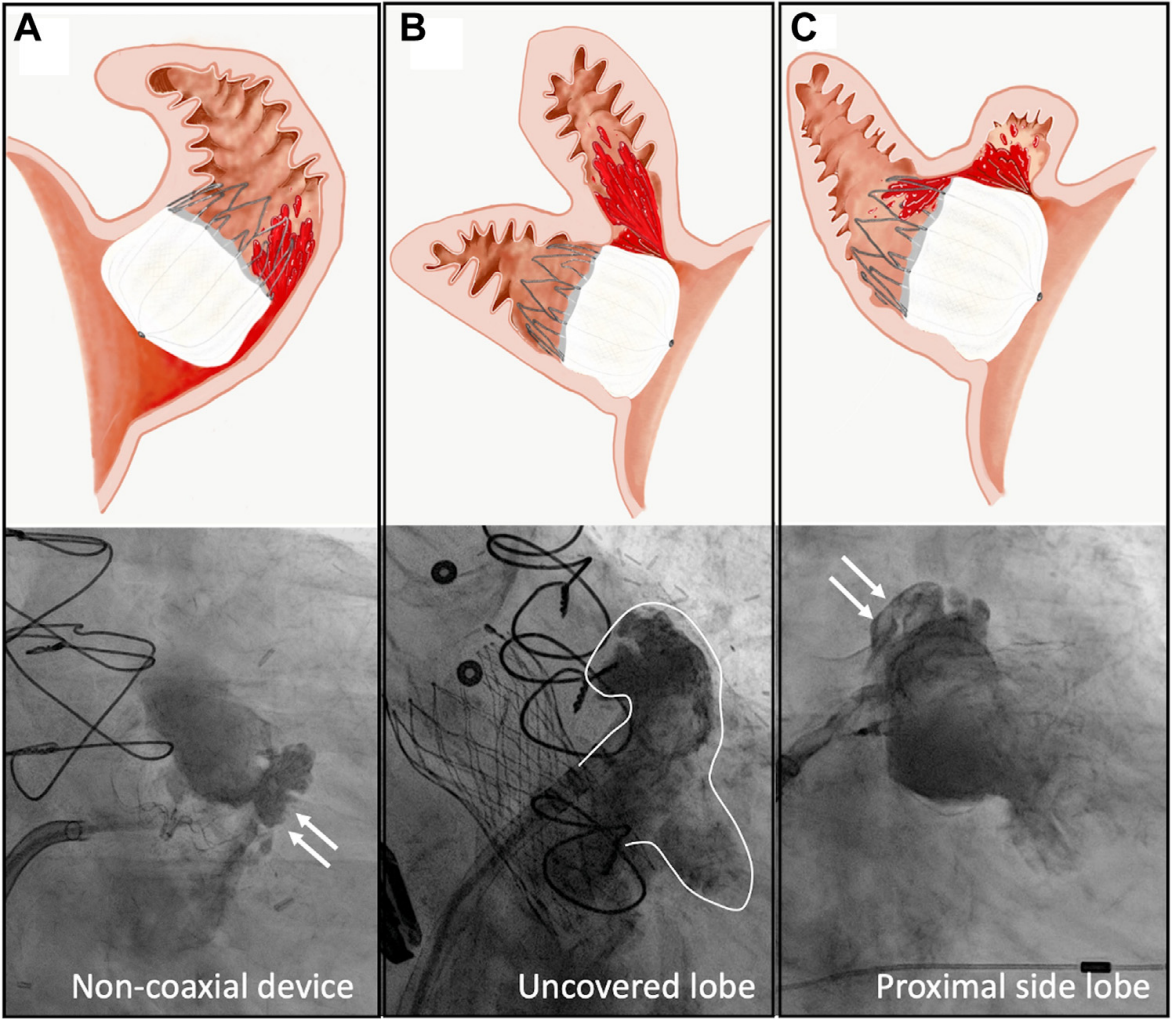
Mechanism of Peri-Device Leak After Left Atrial Appendage Occlusion With the Watchman Device Illustration of the different mechanisms of peri-device leaks. **(A)** Point to the non-coaxial device **(white arrows)**. **(B)** Uncovered lobe. **(C)** Point to the proximal side lobe **(white arrows)**.

**Figure 8:**
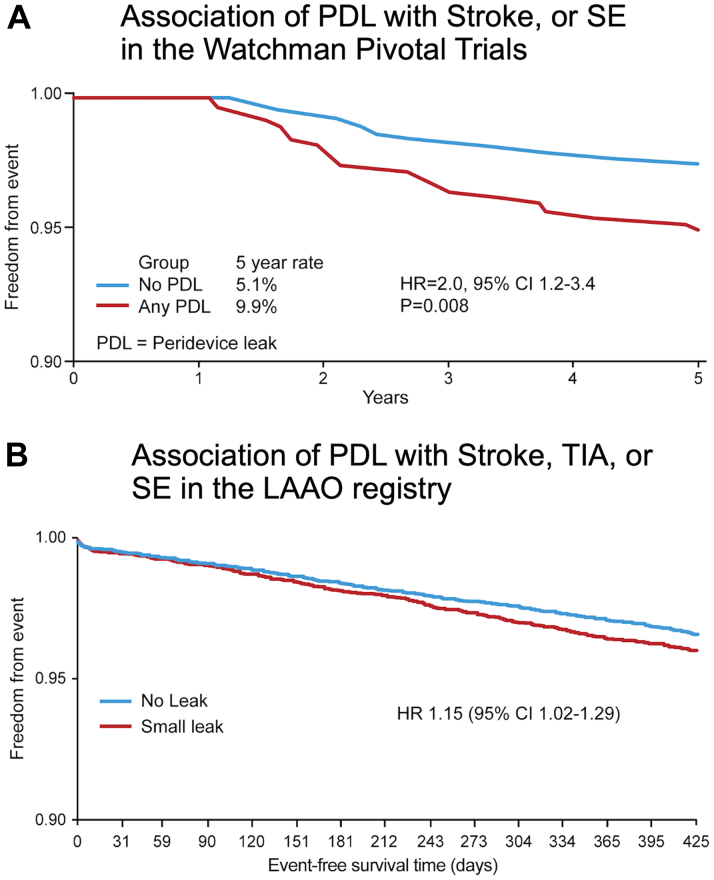
Clinical Impact of Peri-Device Leak After Left Atrial Appendage Occlusion With the Watchman Device **(A)** Data from the Pivotal Trial (PROTECT AF, PREVAIL, and CAP-1). **(B)** Data from the NCDR LAAO Registry. CI = confidence interval; HR = hazard ratio; LAAO = left atrial appendage occlusion; PDL = peridevice leak; PREVAIL = Evaluation of the WATCHMAN Left Atrial Appendage (LAA) Closure Device in Patients With Atrial Fibrillation Versus Long Term Warfarin Therapy); PROTECT AF = WATCHMAN Left Atrial Appendage System for Embolic PROTECTion in Patients With Atrial Fibrillation; SE = systemic embolization; TIA = transient ischemic attack.

Studies attempting to assess whether PDL is associated with a negative impact on clinical outcomes were challenged by several important limitations. First, the rate of stroke or systemic embolization following LAAO is low, and hence, exploring the independent impact of PDL on outcomes requires a very large sample size. Second, the definition of significant vs insignificant leak varies between sites, relies mostly on arbitrary cutoffs of the leak diameter (eg, >3 mm, >5 mm), and does not consider the various mechanisms of the PDL (Figure 7).^47^^,^^48^ Third, there is a wide variability in the assessment and classification of PDL in clinical practice. Fourth, patients with large leaks are currently recommended to remain on anticoagulation, and hence, assessing the differential impact of the residual leak on outcomes in these patients is confounded by a major treatment bias. Therefore, until recently, all published studies that explored this question concluded that PDLs were not associated with thromboembolic events.^33^^,^^45^^,^^49^^,^^50^ Nonetheless, 2 recently presented studies have challenged this assumption. The first is an analysis from the NCDR LAAO registry that documented an association between small leaks (defined as those <5 mm) detected at 45 days after LAAO and major adverse events (driven by ischemic stroke and transient ischemic attack) through 1 year (HR: 1.15; 95% CI: 1.02-1.29).^16^ The second is a long-term analysis from PROTECT-AF and PREVAIL trials and the continuous access to PROTECT AF-2 prospective registry.^51^ In this analysis, small leaks (<5 mm) detected at 1 year were significantly associated with stroke/systemic embolization (9.9% vs 5.1%, *P* = 0.008) (Figure 8).

### Management of PDL

The recent data on PDL suggested that the commonly encountered PDL may not be benign and carries a hazard of major adverse events. However, these studies offered no further insights into the ideal management strategies of PDLs. The current literature of PDL management is sparse. Techniques to minimize PDLs have been described, but data on their impact on PDL mitigation are limited. For example, the role of preprocedural CCT and simulation software to achieve optimal device sizing and coaxial alignment with the LAA has been advocated to achieve a better LAA seal, but the effectiveness of this approach has not been thoroughly studied. Only 1 study to date suggested a potential positive impact of routine preprocedural CCT on reducing the risk of PDL after LAAO. The emergence of various occluder devices with enhanced sealing mechanisms and the availability of steerable delivery sheaths may further enhance the operator’s ability to attain complete closure of the LAAO although studies supporting this assumption remain necessary.

Closure of PDL with coils, plugs, and occluders has been reported in several case series.^48^^,^^52^, ^53^, ^54^ Although these studies showed that complete or near-complete obliteration of the leak is feasible in >90% of patients with low complication rates, the long-term efficacy of this approach is unknown.^49^ Watchful waiting has been proposed as a potential strategy for patients with smaller PDLs due to the documented regression of leaks <5 mm in 20% to 40% of patients.^33^^,^^45^^,^^55^ Whether this is a true leak regression due to atrial remodeling or whether it represents variations in imaging acquisition and interpretation is uncertain. It might be reasonable to observe these patients with repeated imaging considering the limited safety and efficacy data on the alternative approaches such as resumption of anticoagulation or interventional leak closure.

## Procedural optimization

Data from the NCDR LAAO registry documented excellent procedural outcomes with early commercial experience with LAAO in the U.S., including an implantation success of >98% and major complication rate of 2.2%.^17^ Yet, opportunities for further improvement remain considering the preventative nature of the procedure.

### Procedural volumes

Contrary to other structural heart interventions, there are no specific institutional requirements to starting an LAAO program besides having surgical backup on site.^56^ Hence, the number of hospitals and physicians performing LAAO exceeded 490 and 1,100, respectively, within 2 years after the FDA approved the procedure.^21^ During the same period, the median annual institutional and operator volume was 30 and 12 LAAO procedures, respectively. Similar to what is shown with other transcatheter interventions, several studies have shown the importance of maintaining adequate operator experience to achieve optimal outcomes. Nazir et al^57^ showed that a low procedural volume (<15 per year) was associated with a 2-fold increase in major adverse events. Jung et al^19^ suggested a threshold of 32 cases per institution are needed to attain procedural proficiency. Most recently, data from the Amulet IDE trial revealed that the higher rate of pericardial effusion in the Amulet device arm was driven by the inexperience of U.S. operators with the device, suggesting that the impact of operator’s experience on safety outcomes may be device-specific.^58^ With the rapid growth in the number of hospitals and operators performing LAAO and the number of available LAAO devices, there is a need to define the appropriate general and device-specific LAAO experience at both the hospital and the individual operator level to ensure the continuous safety and efficacy of the procedure. Furthermore, there is a need to collate site-specific performance metrics beyond procedural complications (eg, quality of shared decision-making and the adequacy of LAA closure) to better evaluate LAAO programs.

### Procedural planning

**Figure 9:**
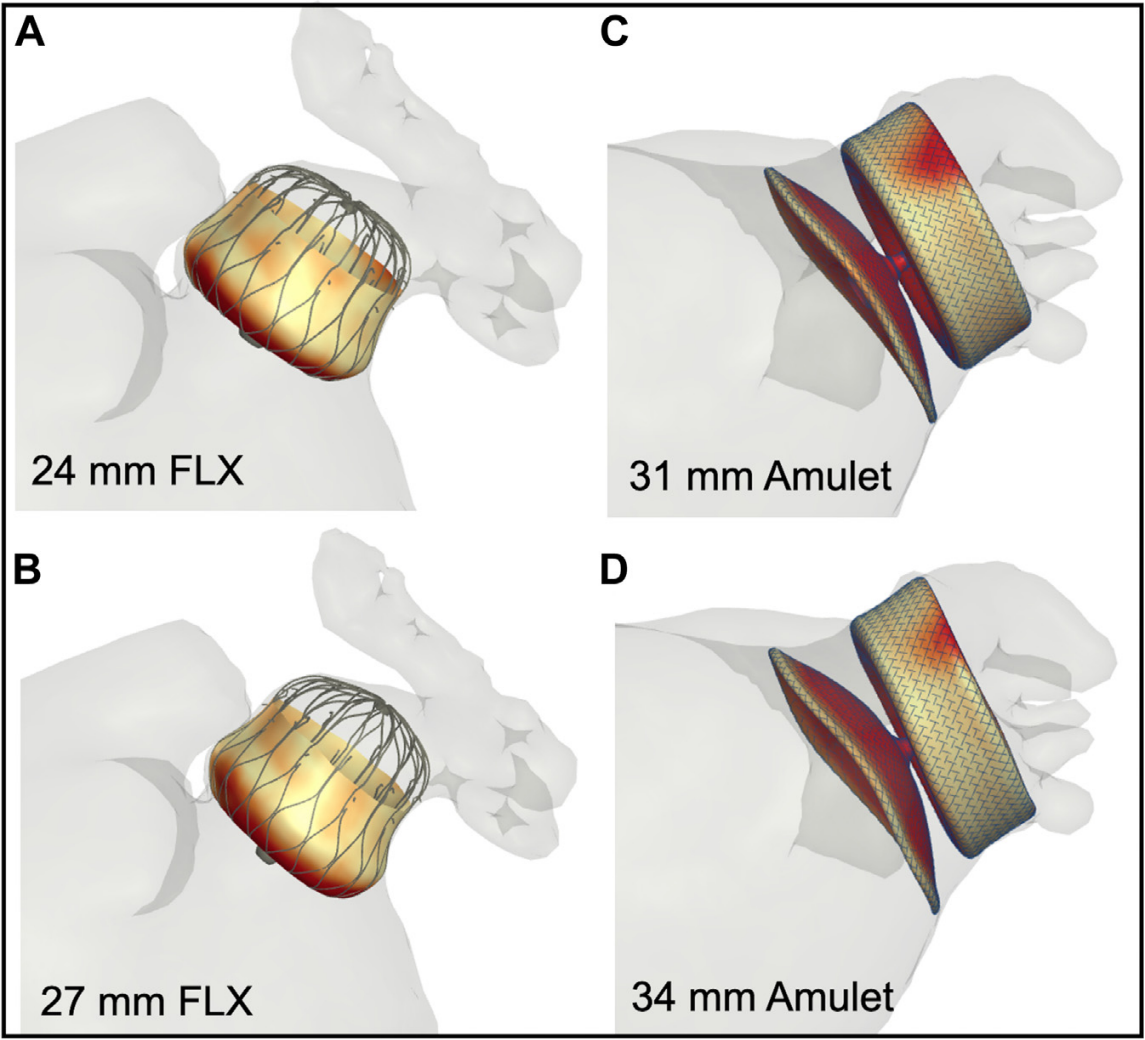
Computational Modeling for Optimization of Left Atrial Appendage Occluder Implantation Illustration of the impact of device size and deployment location of leaks in 2 patients, one treated with the Watchman FLX device **(A, B)** and one treated with the Amulet device **(C, D)**.

TEE is considered the gold-standard modality to assess the size and shape of the LAA prior to the procedure. However, it has now been repeatedly shown that CCT provides a more accurate assessment of the LAA, its geometry, and its dimensions.^59^, ^60^, ^61^ Yet, CCT remains underutilized due to the lack of a standardized methodology in acquiring and interpreting the computed tomography images.^62^, ^63^, ^64^ Nonetheless, a contemporary software program not only provides a user-friendly platform to assess the LAA sizing but also allows virtual implantation of various devices to assess the location, seal, and compression with different LAAO approaches (Figure 9). This has been shown to improve device selection, reduce the number of implantation attempts, and improve procedural time.^65^ Furthermore, recent studies have shown that CCT is more sensitive than TEE in the detection of postprocedural device complications such as DRT or PDL.^62^^,^^66^ Albeit speculative, CCT may soon become the imaging tool of choice for pre- and post-LAAO assessments with easy-to-use machine learning-enabled interactive platforms that can be embedded in the routine workflow of the LAAO practice.^67^, ^68^, ^69^

### The minimalist approach

Akin to what has been observed with transcatheter aortic valve replacement, there is a growing adoption of a minimalist approach to LAAO. This has manifested with the rising interest of intracardiac-echo (ICE)-guided LAAO, the emergence of contrast-free LAAO, and the increasing trends for same-day discharge following the procedure.

#### ICE-guided LAAO

**Figure 10:**
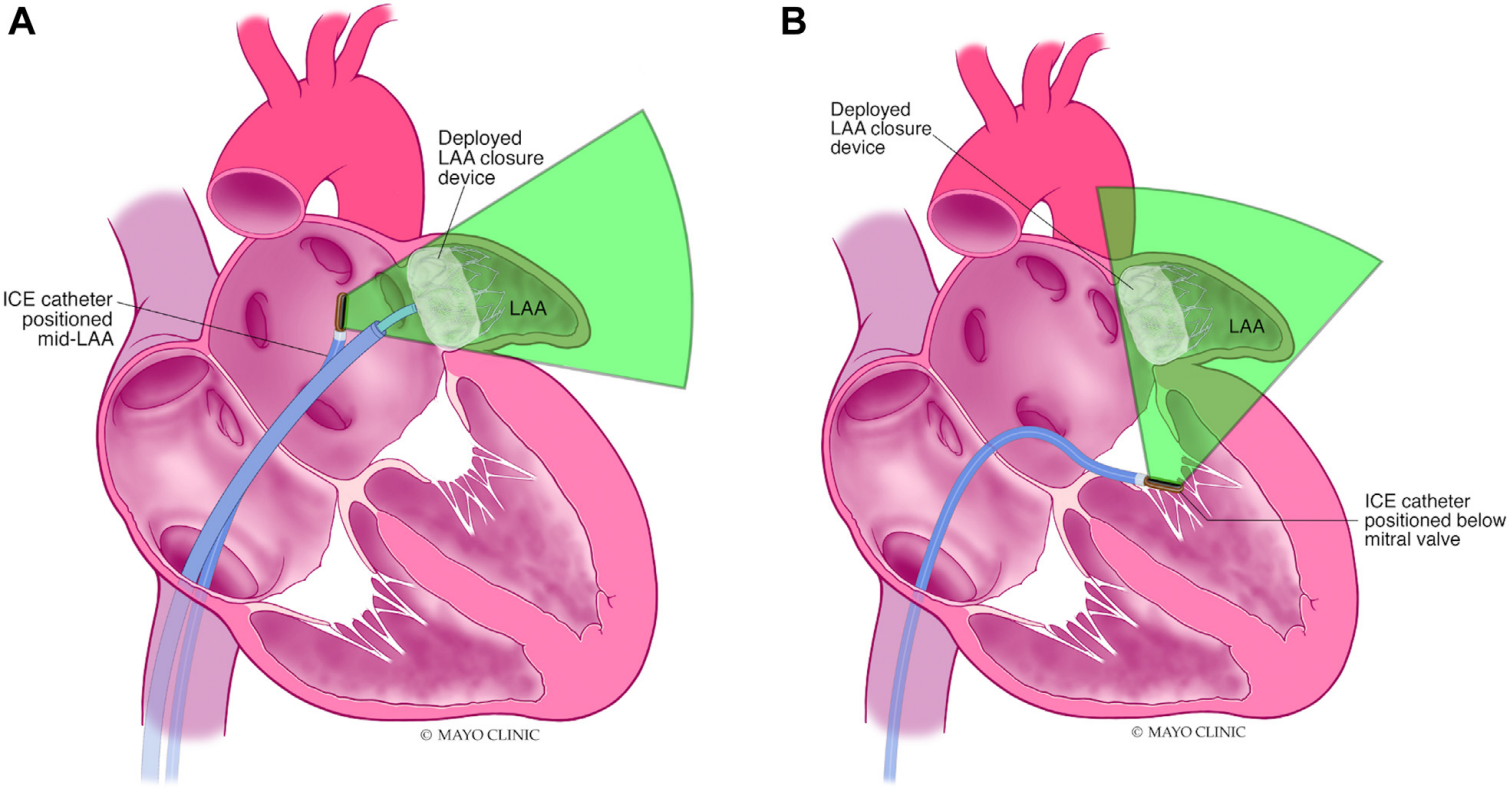
Simplified Imaging Protocol for ICE-Guided LAAO Contrary to the 4 traditional views obtained with transesophageal echo (0, 45, 90, 135 degrees), assessing LAAO with ICE can be adequately achieved with imaging obtaining 2 orthogonal views from 2 locations (mid left atrium and across the mitral valve). **(A)** Mid-left atrial view; **(B)** transmitral view. ICE = intracardiac echocardiogram; LAA = left atrial appendage.

The feasibility for ICE (vs TEE)-guided LAAO has been confirmed in many single-center and multicenter observational studies.^70^ Yet, the adoption rate of ICE in U.S. LAAO practices remained low primarily due to the associated learning curve, the limitations of 2D-ICE, the limited offering of formal educational programs, and the few consensuses regarding the optimal methodologies for imaging acquisition, interpretation, and reporting.^71^ Efforts to validate simple and effective ICE imaging techniques are underway (Figure 10). In addition, the advent of novel 3D- and 4D-ICE technologies has transformed intraprocedural imaging, refueling the interest in ICE-guided LAAO especially during the COVID-19 pandemic.^72^, ^73^, ^74^, ^75^ With the ongoing advancements in the ICE technology, it is anticipated that ICE will become a key imaging modality for procedural guidance in a growing number of LAAO cases worldwide.

#### Same-day discharge

**Figure 11:**
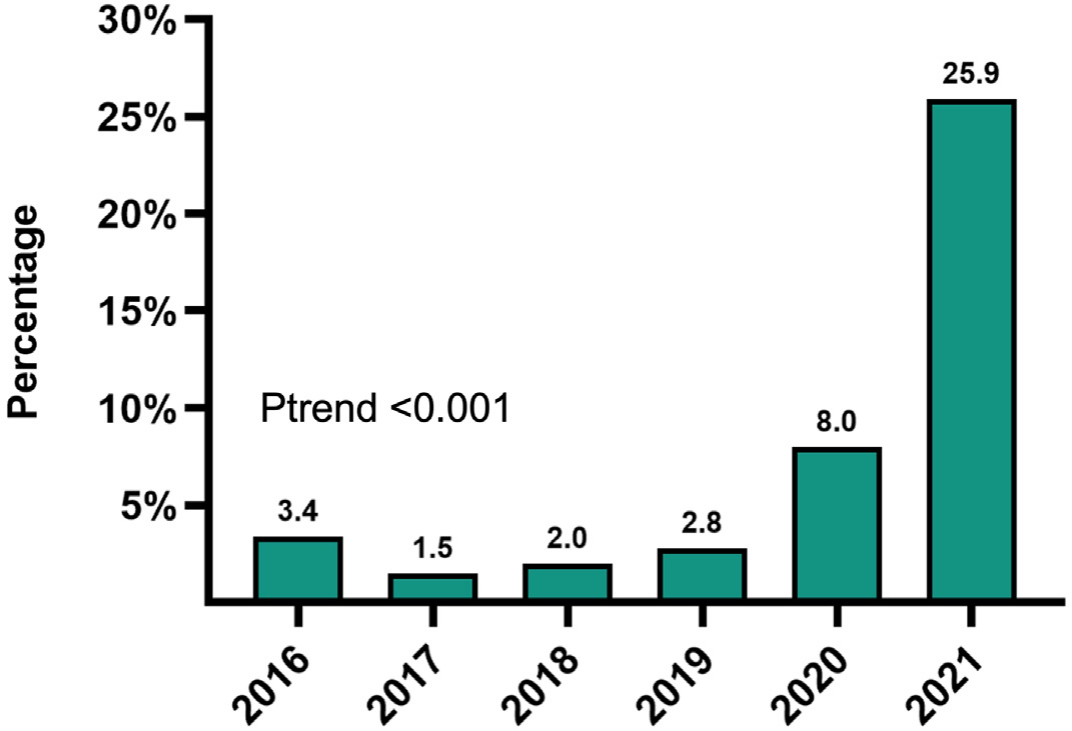
Trends in Same-Day Discharge After Left Atrial Appendage Occlusion Data from Vizient Clinical Database (n = >45,000).

As the safety of LAAO continues to improve, the interest in optimizing resource utilization and cost-effectiveness of the procedure continues to grow. Tan et al^76^ demonstrated the safety and feasibility of same-day discharge after LAAO regardless of the imaging modality utilized (ICE vs TEE). This approach has also been shown to reduce the cost of the LAAO procedure by 15%.^77^ Recent data from a large sample of academic centers in the US revealed a rapid uptake in same-day discharge in the last 2 years with rates approaching 25% of all cases in 2021 (Figure 11).

#### Contrast-less LAAO

**Figure 12:**
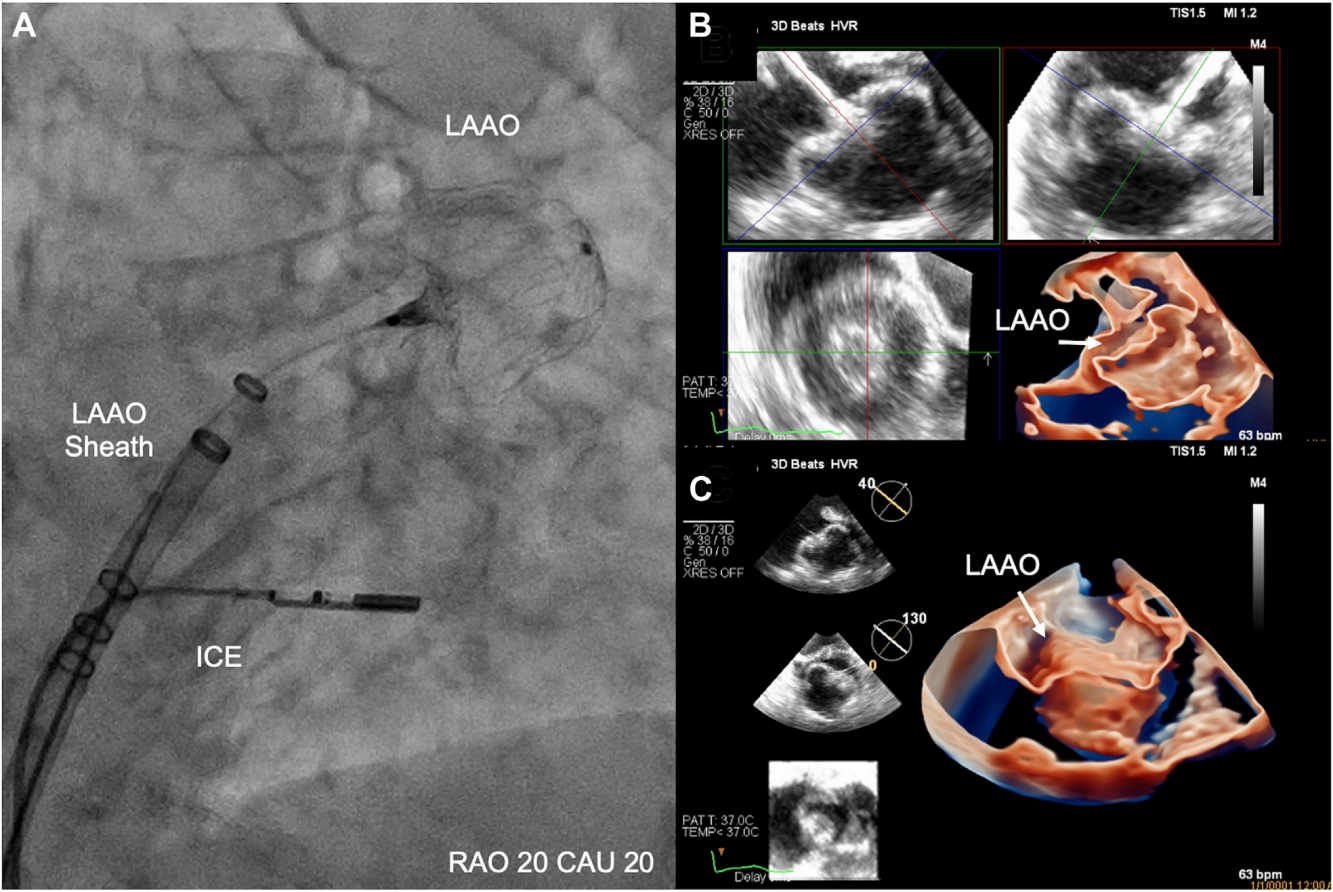
Contrast-Free LAAO Using a Novel 4D-ICE Probe **(A)** Shows the location of ICE probe on fluoroscopy during deployment. **(B**, **C)** Shows the LAA after closure on multiplane 3D imaging from different perspectives. Reprinted with permission from Alkhouli et al. *J Am Coll Cardiol Intv*. 2021:8;14(21):2407-2409. CAU = caudal; ICE = intracardiac echocardiogram; LAAO = left atrial appendage occlusion; RAO = right anterior oblique.

Patients referred for LAAO are usually elderly and have a high (15%-25%) prevalence of chronic renal insufficiency.^17^^,^^78^, ^79^, ^80^ Hence, these patients are at risk of developing acute kidney injury (AKI) after the procedure, which has been shown to carry major negative prognostic implications. In 1 study, the incidence of AKI after LAAO was 9%, and this was associated with a 2.5-fold increase in all-cause mortality at 18 months.^80^ In another study, AKI after LAAO was associated with a 60% higher readmission rate at 6 months.^79^ Hence, efforts have been made to optimize iodine contrast usage in the procedure to mitigate the risk of AKI. Proof-of-concept studies have shown the utility of contrast-free LAAO aided by 3D TEE, ICE, or 3D ultrasound mapping (Figure 12).^81^^,^^82^ If validated in future studies, contrast-free LAAO can be a promising alternative for selected patients with an advanced kidney disease and those at risk of contrast-induced nephropathy.

## Conclusions

LAAO has become a mainstream strategy to address the unmet needs for stroke prevention in a growing number of patients with nonvalvular AF. Despite its excellent safety profile, concerns remain regarding the limited efficacy data, the growing evidence of adverse long-term sequalae of DRT and PDL, and the need for procedural simplification and optimization. These remaining challenges are being addressed in many clinical and preclinical investigations. The results of these studies will further inform the field about the future of LAAO as a promising stroke-prevention modality.

## Funding support and author disclosures

Dr Alkhouli has served on the advisory board for and received research grant support (institutional) from 10.13039/100008497Boston Scientific and 10.13039/100004320Philips; and has received consultation fees from Abbott and Biosense Webster. Dr Ellis is on the advisory board for Atricure, Abbott Medical, Boston Scientific, and Medtronic; and has received research grant (institutional) from 10.13039/100008497Boston Scientific, 10.13039/100004374Medtronic, and 10.13039/100001003Boehringer-Ingelheim. Dr Daniels is on the advisory board of and has received speaker fees from Abbott and Bristol Myers Squibb. Dr Coylewright has received honoraria and research funding from 10.13039/100006520Edwards LifeSciences and 10.13039/100008497Boston Scientific; and honoraria from W.L. Gore. Dr Nielsen-Kudsk is a consultant/proctor for Abbott and Boston Scientific. Dr Holmes has reported that he has no relationships relevant to the contents of this paper to disclose.
